# Robust data‐driven identification of risk factors and their interactions: A simulation and a study of parental and demographic risk factors for schizophrenia

**DOI:** 10.1002/mpr.1834

**Published:** 2020-06-10

**Authors:** David Gyllenberg, Ian W. McKeague, Andre Sourander, Alan S. Brown

**Affiliations:** ^1^ Department of Child Psychiatry University of Turku Turku Finland; ^2^ Department of Adolescent Psychiatry University of Helsinki and Helsinki University Central Hospital Helsinki Finland; ^3^ Welfare Department National Institute for Health and Welfare Helsinki Finland; ^4^ Department of Biostatistics Columbia University Mailman School of Public Health New York New York USA; ^5^ Department of Child Psychiatry Turku University Central Hospital Turku Finland; ^6^ Department of Psychiatry College of Physicians and Surgeons of Columbia University and New York State Psychiatric Institute New York New York USA; ^7^ Department of Epidemiology Columbia University Mailman School of Public Health New York New York USA

**Keywords:** data‐driven, epidemiology, interaction, risk factors, schizophrenia

## Abstract

**Objectives:**

Few interactions between risk factors for schizophrenia have been replicated, but fitting all such interactions is difficult due to high‐dimensionality. Our aims are to examine significant main and interaction effects for schizophrenia and the performance of our approach using simulated data.

**Methods:**

We apply the machine learning technique elastic net to a high‐dimensional logistic regression model to produce a sparse set of predictors, and then assess the significance of odds ratios (OR) with Bonferroni‐corrected *p*‐values and confidence intervals (CI). We introduce a simulation model that resembles a Finnish nested case–control study of schizophrenia which uses national registers to identify cases (*n* = 1,468) and controls (*n* = 2,975). The predictors include nine sociodemographic factors and all interactions (31 predictors).

**Results:**

In the simulation, interactions with OR = 3 and prevalence = 4% were identified with <5% false positive rate and ≥80% power. None of the studied interactions were significantly associated with schizophrenia, but main effects of parental psychosis (OR = 5.2, CI 2.9–9.7; *p* < .001), urbanicity (1.3, 1.1–1.7; *p* = .001), and paternal age ≥35 (1.3, 1.004–1.6; *p* = .04) were significant.

**Conclusions:**

We have provided an analytic pipeline for data‐driven identification of main and interaction effects in case–control data. We identified highly replicated main effects for schizophrenia, but no interactions.

## 1 INTRODUCTION

Evidence suggests that schizophrenia is a disorder with a multifactorial etiology. While individual environmental risk factors for schizophrenia have been identified (Radua et al., 2018), reviews have suggested that the disorder likely results from interactions between them and susceptibility genes (Cannon et al., 2003; van Os, Kenis, & Rutten, 2010). Evidence of interactions can increase the understanding of the disease, and facilitate identifying subgroups at particularly high risk (Zammit, Wiles, & Lewis, 2010).

A substantial number of studies have assessed gene–environment (G–E) interactions in the risk of schizophrenia (Misiak et al., 2018), but critical evaluations have raised doubts whether many findings on such interactions in psychiatry are robust and replicable (Dick et al., 2015; Duncan & Keller, 2011). Apart from G–E interactions, over 30 interactions that do not include specific genetic information have been linked to schizophrenia or psychoses (Table 1). For example, psychotic disorders were associated with different measures of familial psychopathology interacting with urbanicity (van Os et al., 2003; van Os et al., 2004) and maternal infections during pregnancy (Blomström et al., 2016; Clarke et al., 2009) as well as male sex interacting with maternal stress during pregnancy (Fineberg et al., 2016; van Os & Selten, 1998). However, with the exception of the above‐mentioned interactions that were reported in more than one study, we identified no replications over a two‐decade period (Table 1). Possible explanations for the few replications include publication bias, variation in additive versus multiplicative scales to study interactions, and a scarcity of study samples with sufficient sample size and available data to replicate interactions. Nonetheless, it also raises the question regarding the rationale for selecting interactions from a myriad of possibilities. That is to say, even if a study includes only a few variables but all interactions are examined as predictors, one has to decide which predictors or interactions are sufficiently important that they are worthy of analysis.

**TABLE 1:** Summary of reported interactions in the risk of schizophrenia and nonaffective psychoses

Interactions reported in ≥2 papers and interaction in same direction	
Familial liability ✕ urbanicity	
Family history of psychoses ✕ urbanicity (van Os, Hanssen, Bak, Bijl, & Vollebergh, 2003)^a^	
Family history of schizophrenia‐spectrum disorders ✕ urbanicity (van Os, Pedersen, & Mortensen, 2004)^b^	
Family history of any psychiatric hospitalization ✕ urbanicity (van Os et al., 2004)^b^	
Familial liability ✕ maternal infection	
Parental psychosis ✕ prenatal effect of pyelonephritis (Clarke, Tanskanen, Huttunen, Whittaker, & Cannon, 2009)^b^	
Maternal psychiatric disorders ✕ maternal infection during pregnancy (Blomström et al., 2016)^a^	
Male sex ✕ maternal stress during pregnancy	
Male sex ✕ maternal stress during second trimester (van Os & Selten, 1998)^b^	
Male sex ✕ maternal daily stress during pregnancy (Fineberg et al., 2016)^a^	
Interactions reported in ≥2 papers but interaction in opposite direction	
Familial liability ✕ paternal age	
Absent family history of schizophrenia ✕ advanced paternal age (Sipos et al., 2004)^b^	
Sister with schizophrenia‐related diagnosis ✕ advanced paternal age (Perrin et al., 2010)^a^ ^,^ ^c^	
Interactions not found in ≥2 papers	
Familial liability ✕ other risk factor	
Maternal schizophrenia‐spectrum disorder ✕ low family functioning (Tienari et al., 2004)^a^	
Maternal psychosis ✕ unwanted pregnancy (McNeil et al., 2009)^a^	
Biological parent with psychosis ✕ parental employment (Wicks, Hjern, & Dalman, 2010)^a^	
Parental psychosis ✕ maternal depressed mood during pregnancy (Mäki et al., 2010)^b^	
Parental psychosis ✕ high birth weight (Keskinen et al., 2013)^b^	
Parental psychosis ✕ high birth length (Keskinen et al., 2013)^b^	
Parental psychosis ✕ high maternal education (Keskinen et al., 2013)^b^	
Absence of parental psychiatric disorder ✕ parental separation (Paksarian, Eaton, Mortensen, Merikangas, & Pedersen, 2015)^b^	
Parental psychosis ✕ delayed development of touching thumb with index finger (Keskinen et al., 2015)^b^	
Absence of parental psychiatric disorder ✕ childhood residential mobility (Paksarian, Eaton, Mortensen, & Pedersen, 2015)^b^	
Genetic liability ✕ IQ (Kendler, Ohlsson, Sundquist, & Sundquist, 2015)^b^ ^,^ ^d^	
Interactions between other risk factors	
Male sex ✕ refugee status (Hollander et al., 2016)^a^	
Birth year ✕ season of birth (Suvisaari, Haukka, Tanskanen, & Lönnqvist, 2000)^b^	
Birth year ✕ urbanicity (Suvisaari et al., 2000) ^a^	
Season of birth ✕ urbanicity (Harrison et al., 2003)^a^	
Normal Apgar scores at 1 min ✕ advanced paternal age (Sipos et al., 2004)^b^	
Cannabis use ✕ low IQ (Zammit, Lewis, Dalman, & Allebeck, 2010)^a^ ^,^ ^d^	
Cannabis use ✕ poor social relationships (Zammit, Lewis, et al., 2010)^a^ ^,^ ^d^	
Cannabis use ✕ disturbed behaviour (Zammit, Lewis, et al., 2010)^a^ ^,^ ^d^	
Low IQ ✕ poor social relationships (Zammit, Lewis, et al., 2010)^a^ ^,^ ^d^	
Low IQ ✕ disturbed behaviour (Zammit, Lewis, et al., 2010)^a^ ^,^ ^d^	
Low IQ ✕ other diagnosis than psychosis at conscription (Zammit, Lewis, et al., 2010)^a^ ^,^ ^d^	
Obstetric complications ✕ delayed attainment of developmental milestones (Clarke et al., 2011)^b^	
Maternal infection during pregnancy ✕ childhood infections (Blomström et al., 2016)^a^	
Change in degree of urbanicity during childhood ✕ IQ (Toulopoulou, Picchioni, Mortensen, & Petersen, 2017)^b^ ^,^ ^d^	

*Note*: Included studies were published 1998–2017 and reported on an interaction effect in the risk of schizophrenia or related psychoses that did not include specific genetic information. The literature search is described in detail in the Supporting Information.

a The outcome was schizophrenia‐spectrum disorders or nonaffective psychoses.

b The outcome was schizophrenia.

c The finding was restricted to females.

d The study was restricted to males.

The selection of interactions is typically based on theory, resulting in the study of only a fraction of possible interactions. Therefore, important interactions might not be studied, negative findings might not be reported, and findings are less likely to be replicated. To overcome these disadvantages, a data‐driven approach is needed. Certain supervised machine learning techniques allow for addressing such high‐dimensional data by automatically selecting the most informative predictors (Huys, Maia, & Frank, 2016; Iniesta, Stahl, & McGuffin, 2016; Shatte, Hutchinson, & Teague, 2019). While many machine learning techniques are considered to be “black boxes” with low interpretability (Adkins, 2017), the data‐driven selection algorithm “elastic net” has the advantage of producing interpretable results. These algorithms have recently been used to select predictors of antidepressant response (Chekroud et al., 2016), persistence of depression (Kessler et al., 2016), suicide (Kessler et al., 2017) and psychosis (Fusar‐Poli et al., 2016); however, these studies have aimed to develop a predictive model that assigns a probability score for each individual (probability of recovery, suicide, psychosis, etc.), but this does not allow one to draw conclusions about the p‐values and confidence intervals (CI) of the automatically selected predictors. The latter is referred to as post‐selection inference and aims to correct for the large number of predictors present before the selection (Taylor & Tibshirani, 2015). To explore novel high‐dimensional predictors but also ensure that predictors are identified correctly, the false positive (FP) rate needs to be low and the power needs to be high, that is, the approach should have both low Type I and II errors.

Our primary aim was to provide a robust data‐driven approach that allows to study and assess the power of binary main and interaction effects in case–control data. Second, using this analytic pipeline, we tested if replicated parental and demographic risk factors or any of all their possible interactions were associated with schizophrenia. To obtain a large sample size suitable for studying interactions, we utilized a nationwide nested case–control study of schizophrenia in Finland. We included parental and demographic risk factors that have been replicated for this disorder according to a recent umbrella review (Radua et al., 2018), a number of potential confounders, three previously reported interactions between the risk factors (Harrison et al., 2003; Perrin et al., 2010; Sipos et al., 2004; van Os et al., 2003; van Os et al., 2004) but also all other possible interactions.

## 2 METHODS

**FIGURE 1:**
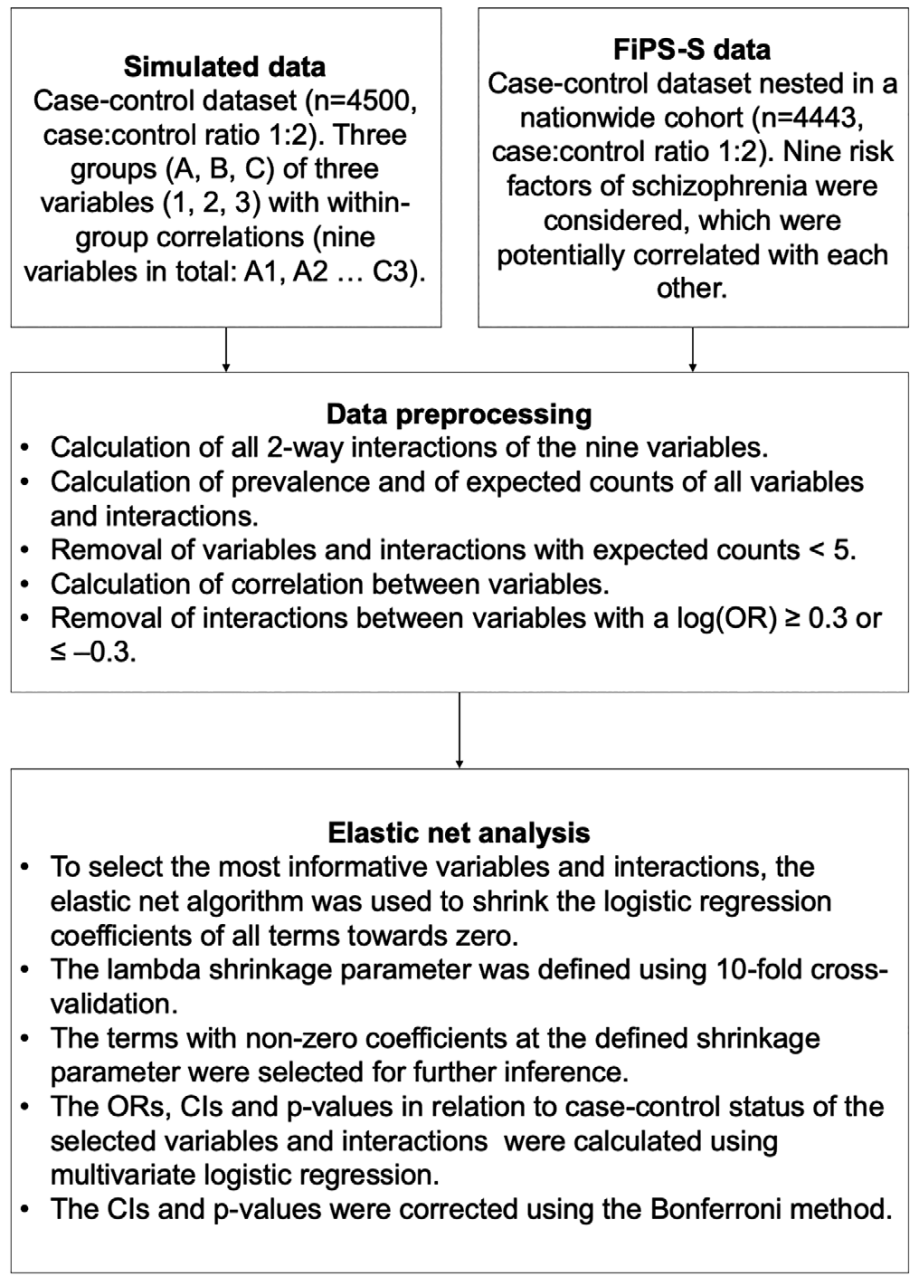
Flow‐diagram showing the analytic pipeline

The study was based on both a simulation study and actual data from the Finnish Prenatal Study of Schizophrenia (FiPS‐S) which utilized a nested case–control design (Gyllenberg et al., 2016). A schematic overview of the methods used in both the simulation and the FiPS‐S study is shown in Figure 1. The analytic pipeline and the simulation study are described in detail under “Analytic pipeline,” and the FiPS‐S is described below.

### 2.1 Design of the FiPS‐S study

The source population consisted of all subjects born in Finland between 1983 and 1998 (*n* = 1,009,846; Statistics Finland, 2018). We used nationwide registers to identify all cases who had been diagnosed with schizophrenia or schizoaffective disorder before December 31, 2009 (maximum age = 26 years) and randomly selected age‐ and sex‐matched controls (see “Case and Control Identification”). The study was approved by the ethical committees of the Hospital District of Southwestern Finland, by the National Institute of Health and Welfare, and the Institutional Review Board of the New York State Psychiatric Institute.

### 2.2 Description of registers

To identify subjects and their parents and to obtain information on predictors, the personal identification number (PIN) was used and linked to national registers. The PIN has been assigned to all residents by the Finnish Population Register since 1971. The computerized nationwide Finnish Population Register (FPR) was established in 1971, was used to retrieve data on place of birth, date of emigration, date of death, and biological parents, including their dates of births. To identify all recorded diagnoses for psychiatric hospital admissions and psychiatric outpatient treatment visits among study subjects and their parents, we used the Finnish Hospital and Outpatient Discharge Register (FHDR) which is maintained by the National Institute of Health and Welfare. The FHDR was established in 1963; computerized data are available since 1987 and the register includes all public outpatient visits since 1998. The register contains the personal and hospital identification codes and primary/secondary psychiatric diagnoses. Finally, Statistics Finland was used to obtain data on parental education level at birth.

### 2.3 Case and control identification

We identified all cases with schizophrenia (ICD‐10 code F20) or schizoaffective disorder (F25) from the FHDR. For brevity, we use “schizophrenia” for referring to schizophrenia or schizoaffective disorder. The diagnostic validity of schizophrenia in the FHDR from a previous study showed that 93% of subjects with a diagnosis of schizophrenia in the FHDR were assigned a consensus diagnosis of schizophrenia (Mäkikyro et al., 1998). Controls were selected from the source population; they were without schizophrenia, other nonaffective psychotic disorders, or bipolar disorder; and were matched on the date of birth (±1 month), for sex, and residency in Finland at the time of case diagnosis. There were many controls with these characteristics; therefore, two controls per case were randomly drawn from all controls fulfilling these criteria.

There were 1,505 subjects who had received a schizophrenia diagnosis during the study period and 3,010 age‐ and sex‐matched controls. Subjects with unknown fathers were excluded and, 1,469 schizophrenia cases (97.6%) and 2,975 controls (98.8%) had complete data on all risk factors and were included in the analyses.

### 2.4 Predictors

We included nine risk factors with replicated associations in a recent umbrella review (Radua et al., 2018) and other previous studies (Davies, Welham, Chant, Torrey, & McGrath, 2003; Eaton & Harrison, 2001; El‐Saadi et al., 2004; Heinz, Deserno, & Reininghaus, 2013; Kemppainen et al., 2001; Leung & Chue, 2000; McGrath et al., 2014; Petersen, Mortensen, & Pedersen, 2011; Rasic, Hajek, Alda, & Uher, 2014; van Os et al., 2010). Male sex, birth between December and March, urbanicity, low parental education, parental psychosis, paternal age 35 or older (at time of birth of the study subject), maternal age 35 or older, paternal age 19 or younger, maternal age 19 or younger and all their two‐way interactions were assessed. All variables were derived from the FPR, except for parental psychosis, which combined information from the FPR and FHDR. Despite the matching on sex and date of birth, we included male sex and birth period as predictors in order to assess possible interactions including the variables. To maximize the number of subjects with advanced parental age, we set the upper cutoff of parental age to 35 years; this is the age at which the risk of schizophrenia begins to increase according to a meta‐analysis (Wohl & Gorwood, 2007). Urban birth was classified based on national standards used by Statistics Finland (Official Statistics of Finland, 2015): a densely populated area was defined as a 250 m^2^ area with >200 inhabitants; we classified municipalities with ≥90 and <90% of the population living in densely populated areas as urban and nonurban, respectively. Low parental education was defined as both parents not having postelementary school education. Parental psychosis was defined as a lifetime diagnosis of nonaffective psychoses according to the FHDR (see Table S1 for ICD‐codes).

### 2.5 Analytic pipeline

The steps in the analytic pipeline are depicted in Figure 1 and described in detail below. The data preprocessing and all analyses were the same for the simulation datasets and the real data. All analyses were performed with R version 3.6.3. The R code for reproducing all the steps in the analytic pipeline is publicly available at https://github.com/davgyl/dd_ident. As the access to the register‐based health care data is limited and the original data cannot be released, we provide code to reproduce the analyses with simulation data.

#### 2.5.1 Simulation study

The true and FP rates for correctly identifying main and interaction effects were assessed in a simulation study. Each simulated dataset comprised three groups (A, B, C) of three variables (nine variables in all: A1, A2, A3, B1…C3) with a case: control ratio of 1:2.

First, we defined simulated datasets as follows: among 4,500 subjects, we set the prevalence of the variables within each group at 20% for the first variables (A1, B1, C1), 15% for the second variables (A2, B2, C2) and 5% for the third variables (A3, B3, C3); we set a within‐group correlation of 0.3 in the three groups of variables (A, B, C); we set a main effect of OR = 1.3 for the first variable in each group (A1, B1, C3), and we set one active interaction to have OR = 3.0 (A2 ✕ B2); this interaction was taken between variables in uncorrelated groups having prevalence of 15%, so the interaction itself had a prevalence of 2.3%. A simulated dataset using these defintions is visualized in the Supplement (see “R‐code to reproduce analyses”). A detailed rationale for why these definitions were used is described in the Supporting Information.

Second, we defined 13 other types of simulation datasets by exchanging one of the following in the datasets: the number of subjects (9,000 or 15,00 instead of 4,500); the prevalence of the active interaction (2.9% or 4.0% instead of 2.3%); the odds ratios (OR) of the interaction effect (1.5, 2.0, 2.5, 3.5, 4.0, 4.5, or 5.0 instead of 3.0) or the within‐group correlation (0.1 or 0.5 instead of 0.3). Based on 10,000 simulations for each type of dataset (140,000 simulations in total), we calculated the empirical power and FP rate of main and interaction effects.

Third, we conducted additional analyses of simulations with three active interactions, but with no active main effects and with less resemblance to the FiPS‐S data. These analyses are described in the Supporting Information Methods.

#### 2.5.2 Data preprocessing and descriptive analyses

To maximize interpretability of interactions, all main effects were coded as binary (0, 1; Table S1); thus, all two‐way interactions also had a binary structure with “1” indicating presence of both risk factors. To describe the data, we calculated frequencies and proportions of all predictors. To ensure stable models without zero or near‐to‐zero variance, we excluded interactions with a lower expected count of five among cases and controls prior to further modeling. To minimize the risk of falsely identifying interactions that could be explained by high correlation, we further excluded interactions between main effects that had had log(OR)s ≥0.3 or ≤−0.3. The decision to use the above criteria for excluding predictors was informed by a simulation study (Supporting Information Methods). During the preprocessing of data, the prevalence, the expected count and the correlation were extracted for descriptive purposes. Finally, before modeling, the predictors were standardized by scaling and centering, while the original binary structure of the predictors was used for inference. All the preprocessed predictors in the model, that is, interactions were studied on the multiplicative scale.

#### 2.5.3 Elastic net analyses

We performed variable selection using the elastic net algorithm (Zou & Hastie, 2005) using the R package *glmnet* (Friedman, Hastie, & Tibshirani, 2010). Due to the binary outcome (schizophrenia, control), we used the logistic regression version of the algorithm. The algorithm is an extension of generalized linear models and can select the strongest associations by shrinking the regression coefficients of predictors as a function of the shrinkage‐parameter lambda and of the tuning parameter alpha. Low alpha values near zero favor ridge regression and high alpha values near one favor lasso regression; we conducted a simulation study for choosing an optimal alpha value of 0.75 (Supporting Information Methods). As predictors with non‐zero coefficients are defined as the “selected predictors” in elastic net models with an alpha parameter >0, the number of selected predictors is a function of lambda. We defined the lambda parameter using 10‐fold cross‐validation and the 1‐SE‐rule; for more exploratory approaches the minimum‐rule can be used instead.

After selecting the most informative predictors, we calculated their OR, CI, and *p*‐values. We fitted a multivariate logistic regression model of the selected predictors and corrected the Wald‐type CIs and *p*‐values with Bonferroni‐adjustment: the significance level of 5% was diminished by dividing it by the number of all predictors included in the selection process. For example, the elastic net algorithm chose between 31 predictors in the FiPS‐S data, and in that analysis the Bonferroni‐corrected *p*‐values were defined as *p*‐values divided by 31 and the level of the CI are set at 99.84% (1–0.05/31 = 1–0.0016 = 0.9984). Although not used in the current study, the postselection inference approach also allows for more exploratory analyses with less strict significance correction and more narrow CI by setting the significance level to, for example, 10% (see “R‐code to reproduce analyses” in the Supporting Information).

#### 2.5.4 Additional exploratory analyses

To compare our analytic approach to traditional marginal screening of main and interaction effects, we conducted simulation studies and marginal screening of previously reported interaction effects (Supporting Information Methods).

## 3 RESULTS

### 3.1 Simulation results

Based on 10,000 simulated datasets for each definition of dataset, the FP rate of detecting active main or interaction effect was <5% regardless of how the datasets were defined (Table 2). However, the true positive (TP) rate, that is, the power, to detect the active main or interaction effects varied considerably depending on the definition of the simulated datasets. As shown in Table 2, using our primary definition of simulated datasets, the power to detect an active main effect was 57.3% and the active interaction was 46.6%, but when the number of subjects were increased from 4,500 to 9,000, the power to detect main and interaction effects exceeded 80%. Furthermore, the power to detect the active interaction was ≥80% when the prevalence of the active interaction was 4.0% instead of 2.3% or when the OR of the active interaction was 5 instead of 3. The degree of within‐group correlation also affected the power to detect the active interaction, but the power remained <80%.

**TABLE 2:** The true positive (TP) and false positive (FP) rates of identifying main and interaction effects in 10,000 simulated datasets using elastic net variable selection and Bonferroni‐corrected multivariate logistic regression

	Definition of simulated datasets				Identification of main effects		Identification of interaction effects	
	OR of active interaction	No. of subjects	Prevalence of active interaction (%)	Within‐group correlation	TP^a^ (%)	FP^b^ (%)	TP^c^ (%)	FP^d^ (%)
Primary definition of simulated datasets	3.0	4,500	2.3	0.3	57.3	1.3	46.6	4.5
Varying OR of active interaction								
	1.5	4,500	2.3	0.3	60.0	1.3	1.7	4.8
	2.0	4,500	2.3	0.3	58.9	1.3	11.3	4.2
	2.5	4,500	2.3	0.3	58.1	1.1	29.1	4.2
	3.5	4,500	2.3	0.3	57.4	1.0	60.3	4.0
	4.0	4,500	2.3	0.3	57.7	1.0	70.0	3.9
	4.5	4,500	2.3	0.3	57.8	1.1	78.1	4.3
	5.0	4,500	2.3	0.3	57.8	0.8	**83.4**	**3.8**
Varying number of subjects								
	3.0	9,000	2.3	0.3	**93.9**	**0.6**	**94.9**	**3.1**
	3.0	15,000	2.3	0.3	**99.8**	**0.5**	**99.7**	**2.0**
Varying prevalence of active interaction								
	3.0	4,500	2.9	0.3	56.6	1.0	67.0	4.2
	3.0	4,500	4.0	0.3	55.5	1.0	**86.9**	**4.0**
Varying within‐group correlation								
	3.0	4,500	2.3	0.1	57.4	0.9	51.2	4.4
	3.0	4,500	2.3	0.5	54.8	1.2	36.2	4.0

*Note*: Simulated datasets with both the TP rate ≥80% and the FP rate <5% are shown in bold.

a The TP rate of identifying main effects was defined as the proportion of simulations in which at least one of the three active main effects were correctly identified.

b The FP rate of identifying main effects was defined as the proportion of simulations in which at least one of the nonactive main effects were incorrectly identified.

c The TP rate of identifying interaction effects was defined as the proportion of simulations in which the one active interaction effect was correctly identified.

d The FP rate of identifying interaction effects was defined as the proportion of simulations in which at least one of the nonactive interactions effects were incorrectly identified.

In an additional simulation study, we set all variables to the have same prevalence, including no active main effects and three active interaction effects (Supporting Information Results). In these analyses, the FP rate remained <5%, the power to identify at least one active interaction ranged between 22.9% and 100.0% depending on the effect size of the active interactions, and the power to identify all three active interaction effects ranged between 0.1 and 93.4% (Table S3).

We further tested whether our analytic approach was superior to traditional marginal screening of main and interaction effects, that is, testing one predictor at a time without variable selection (Supporting Information Methods and Results). When we did not apply Bonferroni‐correction to marginal screening, the FP rates were high, ranging between 47.1 and 91.1% (Table S4). When we applied Bonferroni‐correction to marginal screening, the TP and FP rates for detecting interactions were similar to our approach of elastic net variable selection, but the FP rate for identifying main effects ranged between 3.6 and 71.1% when using marginal screening (Table S5) compared to between 0.5 and 1.3% when using variable selection (Table 2).

### 3.2 Results based on FiPS‐S data

**FIGURE 2:**
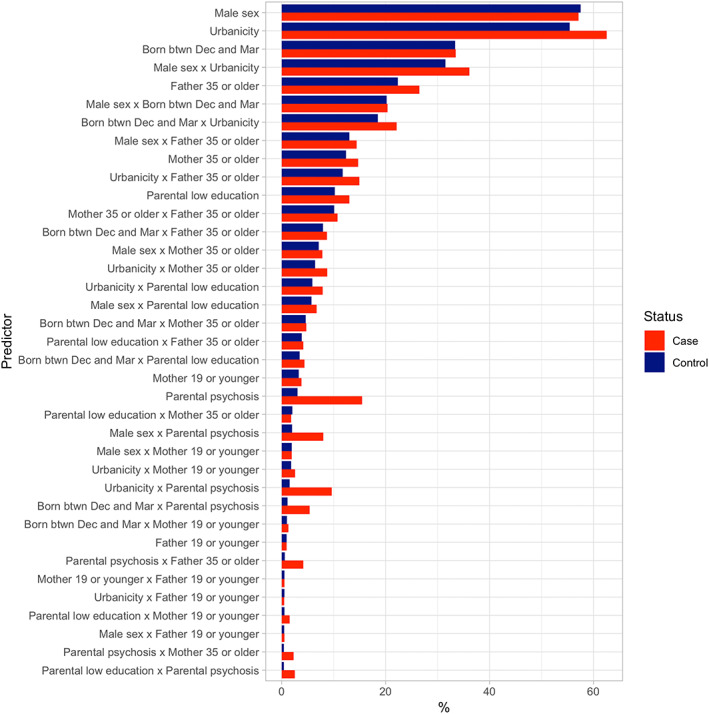
Prevalence of analyzed predictors among schizophrenia cases and controls ranked by percentage in controls. btwn, between; Dec, December; Mar, March

**FIGURE 3:**
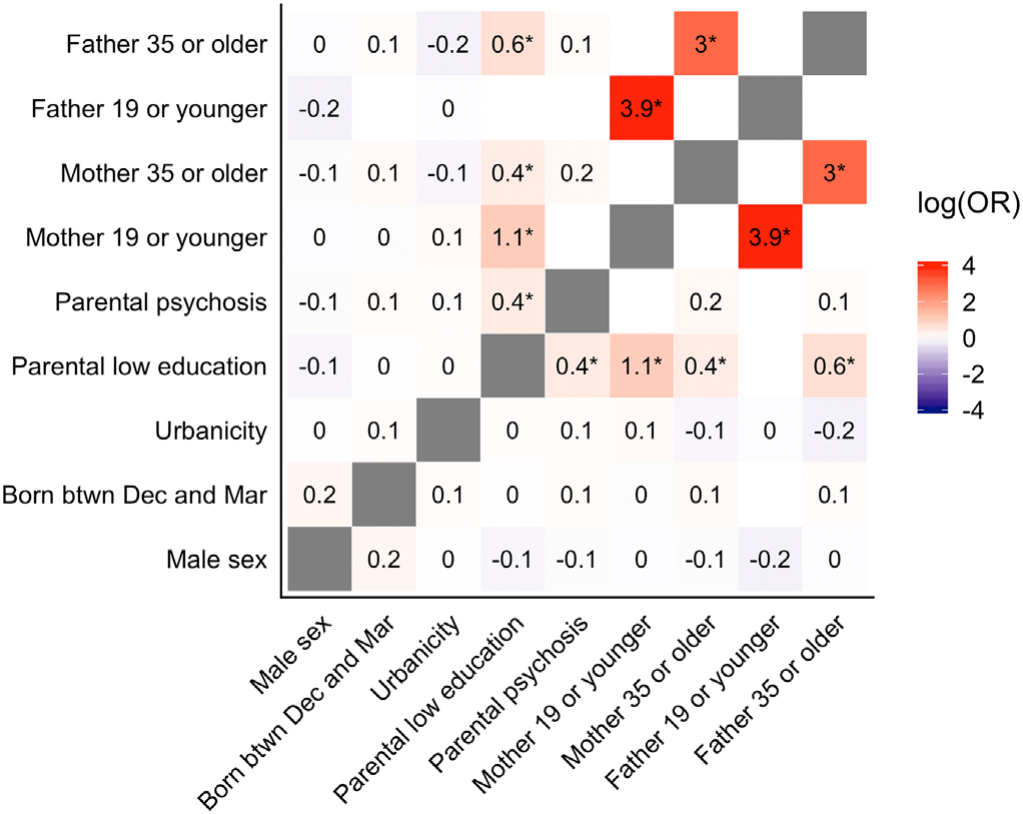
The log(odds ratios [OR]) between main effect variables. The asterisks denote log(ORs) with values ≥0.3 or ≤−0.3. The log(ORs) of variables with expected count <5 were not assessed and are denoted with blank squares in the heatmap. btwn, between; Dec, December; Mar, March

There were nine main effects and 34 possible interactions between main effects. First, we inspected the frequencies and the expected counts of all these 43 predictors by case–control status and as part of the data preprocessing, we excluded six interactions that had an expected count lower than 5 (Table S6). The prevalence of these 37 main and interaction effects is summarized in Figure 2. Second, we inspected the correlational structure between main effect variables as shown in Figure 3. There were six variables that were associated with each other at log(OR)s ≥0.3 or ≤−0.3 (Figure 3): parental low education with mother 19 or younger, with mother 35 or older, with father 19 or younger and with father 35 or older; mother 19 or younger with father 19 or younger; and mother 35 or older with father 35 or older; the interactions between these variables were excluded from the analyses. In total, 31 main effects and interactions (hereafter “predictors”) were included in the analyses.

**FIGURE 4:**
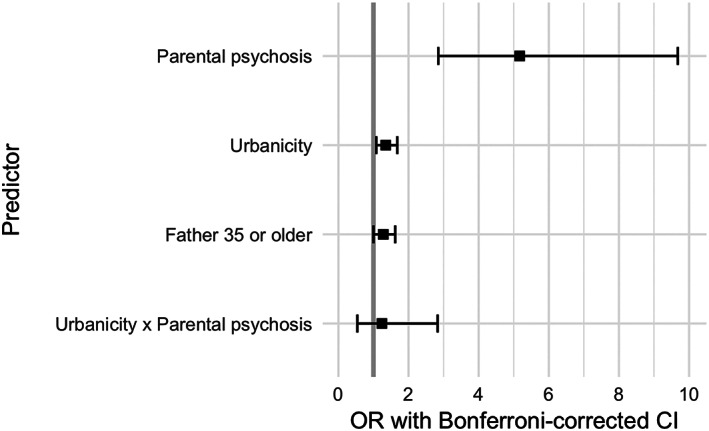
Forest plots of odds ratios (OR) and Bonferroni‐adjusted confidence intervals (CI) of the selected predictors. The gray vertical line corresponds to OR = 1

When using the elastic net algorithm to select the most informative predictors, the following predictors were selected: parental psychosis, urbanicity, father 35 or older and urbanicity ✕ parental psychosis. The respective proportions of cases and controls with the selected predictors were as follows: parental psychosis 15.5 versus 3.1%; urbanicity 62.6 versus 55.4%; father 35 or older 26.5 versus 22.4%; and urbanicity ✕ parental psychosis 9.7 versus 1.5% (Figure 2). Figure 4 shows the ORs and Bonferroni‐corrected CIs from the associations between the four selected predictors and schizophrenia. Associations with schizophrenia were significant for parental psychosis (OR = 5.2, CI 2.9–9.7; *p* < .001), urbanicity (1.3, 1.1–1.7; *p* = .001) and father 35 or older (1.3, 1.004–1.6; *p* = .04). Of note, the interaction between urbanicity and parental psychosis was not significant and no other interactions had been selected for further analysis by the elastic net algorithm.

To further explore whether previously reported interactions could be detected using a less strict approach in the FiPS‐S data, we conducted marginal screening without Bonferroni‐correction of the interactions between parental psychosis and urbanicity, parental psychosis and father 35 or older, and born in winter months and urbanicity; however, none of the interaction terms were significant (Table S7).

## 4 DISCUSSION

This study has two major findings. First, using a simulation study defined to resemble epidemiologic data, the data‐driven approach identified both main and interaction effects with sufficient power and strict control of FPs. However, for that to occur, the number of subjects had to be large, the predictor common or the effect size large. Second, when studying relatively common risk factors and interactions between parental and demographic factors predicting schizophrenia in a national birth cohort, this analysis identified three of the most replicated risk factors for schizophrenia—parental psychosis, urbanicity and advanced paternal age—but none of the studied interactions were associated with schizophrenia.

The highest level of evidence of a risk factor can be obtained by umbrella reviews, followed by meta‐analyses and original research (Fusar‐Poli & Radua, 2018). It is reassuring that our original research with a data‐driven approach selected and showed significant findings explicitly for parental psychosis, urbanicity, and paternal age ≥35 years, which were also significant in a recent umbrella review of risk factors for psychosis (Radua et al., 2018). Although the outcome could be defined as psychosis instead of schizophrenia or the risk ratios could be reported instead of ORs in the umbrella review (Radua et al., 2018), it is of note that the CI of the effect sizes were comparable in our study versus the umbrella review (parental severe mental illness: 3.0–11.8 versus 2.9–9.7; urbanicity: 1.6–3.1 versus 1.1–1.7; and paternal age ≥35 years: 1.06–1.4 versus 1.004–1.6). In other words, the reported significant main effects are replications in line with the literature that strengthen the applicability of our analytic pipeline.

However, the negative findings from our systematic exploration of interactions require reconciliation, as previous studies have reported positive findings (Table 1). First, the analytic approaches used in those studies were fundamentally different as we used a data‐driven approach to examine interactions while other studies were theory‐driven. In a theory‐driven approach, one or a few interactions are typically selected to be tested and no statistical correction for this selection‐process is applied. In data‐driven approaches, it is required to correct for the selection‐process for reducing the risk of FP errors (Taylor & Tibshirani, 2015). We corrected for FPs with the Bonferroni method, and in our simulation study, we showed that in sample sizes like ours the analytic pipeline had <5% FP error rate and ≥80% power to detect interactions with prevalence over 4% and ORs over 3. Theory‐driven studies without statistical correction can detect more rare interactions in smaller samples, but then there should be no exploratory elements in the analyses for maintaining control of FPs. The degree of possible exploratory analyses in previous studies cannot be assessed and we can therefore not draw any conclusions about possible FPs. However, the risks inherent in post hoc interpretations are decreased in theory‐based quasi‐experimental studies, such as adoption studies, as they are designed to study a limited number of interactions. For example, adoption studies have been designed to test interactions between psychosis in a biological parent and adoption family characteristics (Tienari et al., 2004; Wicks et al., 2010). Nonetheless, most other studies, such as population‐based cohorts or family cohorts, have been nonexperimental like the present one, and the differences in design are unlikely explanations for the majority of observed discrepancies. Second, we did not have measurements on many of the variables involved in previously reported interactions, for example, maternal stress during pregnancy (Fineberg et al., 2016; van Os & Selten, 1998). To maximize our sample size, we included variables that were available in the registers during the sampling period. We were able to assess three previously reported interactions, namely between family liability and urbanicity (van Os et al., 2003, 2004), between family liability and advanced paternal age (Perrin et al., 2010; Sipos et al., 2004), and between season of birth and urbanicity (Harrison et al., 2003). The interaction between family liability and urbanicity has been replicated when analysed with multiple degrees of urbanicity on an additive scale (van Os et al., 2003, 2004), but when analysed on a multiplicative scale as in our study, the finding was nonsignificant (Mortensen et al., 1999). The interaction between family liability and advanced paternal age have shown findings in opposite directions (Table 1): one study with family liability present in the interaction (Perrin et al., 2010) and another with family liability absent in the interaction (Sipos et al., 2004). Finally, the interaction between season of birth and urbanicity was significant in a study showing that the hazard ratios of urban birth in relation to nonaffective non‐schizophrenic psychoses were 2.7 for those born in winter and 1.3 for those born in summer (Harrison et al., 2003). The corresponding ORs of urban birth for those born in winter and non‐winter months were 1.6 and 1.3, respectively, in our study. This indicates that our results are in line with the literature, but that no significant interaction between season of birth and urbanicity could be detected even with a traditional approach. In summary, given all these differences, we can neither confirm nor deny the presence of interactions in previous studies.

Our analytic approach avoided FPs successfully and showed suitability of analyzing a large number of predictors, but a drawback of the strict Bonferroni correction is a substantial reduction in exploratory findings in small datasets: low power to detect interactions when the prevalence is below 4% and ORs under 3, even for our large sample. We carried out a simulation to see whether the traditional marginal screening approach of testing one variable at a time yields different results. Without Bonferroni‐correction to marginal screening, the FP rate was over 50%. While we opted for elastic net variable selection with Bonferroni‐correction (due to its strong performance with correlated predictors, and the possibility of obtaining easily interpretable CI and *p*‐values), we also considered a handful of other approaches. For example, the fixed lambda approach allows for postselection inference CI and p‐values using the LASSO penalty (Taylor & Tibshirani, 2018; Tibshirani, Taylor, Lockhart, & Tibshirani, 2016), but has not been developed for the elastic net algorithm which performs better with correlated predictors (Zou & Hastie, 2005). We also considered the recently developed knockoff filter that controls the false discovery rate (Barber & Candès, 2015, 2019), but opted for our approach, consistent with the tradition in psychiatric epidemiology, to control the family‐wise error rate and to report Wald‐type CI. Identifying predictors using these and other novel postselection inference techniques remains an active field of study.

The strengths of this study include several advantages of the FiPS‐S, including prospectively acquired data in a population‐based birth cohort, comprehensive registry‐based information, and the application of novel statistical methods. The following limitations should be considered. First, as noted above, we could not assess many of the previously reported interactions as these were not included in the register. Second, despite the large sample size, our simulation study showed that there is little power to detect rare combinations of risk factors; future studies with larger sample sizes will be required to detect them. Third, in our study the maximum age of schizophrenia was 26 years; future studies with longer follow‐up will be needed to reassess our findings.

## 5 CONCLUSIONS

We provide a data‐driven approach that can be used to detect robust associations in high‐dimensional case–control data. Using this technique, we identified previously replicated risk factors that were significantly associated with schizophrenia, though we did not find support for interactions between the studied risk factors. Clinicians should acknowledge the uncertainty related to nonreplicated interactions and refrain from drawing conclusions about individual patients' risk for schizophrenia based on such findings. A major challenge remains as to how the field can identify interactions and other predictors in high‐dimensional data that replicate across independent samples. Data‐driven approaches with even larger datasets and more detailed variables on neurodevelopment are likely to provide a fruitful way forward to address this challenge.

## CONFLICT OF INTEREST

All the authors report no biomedical financial interests or potential conflicts of interest.

## Supporting information


**Appendix S1**: Supporting information
