# Supplementary material for: Robust data‐driven identification of risk factors and their interactions: A simulation and a study of parental and demographic risk factors for schizophrenia
Source: Int J Methods Psychiatr Res. 2020 Jun 10;29(4):e1834. doi: 10.1002/mpr.1834 (PMC7723216; doi:10.1002/mpr.1834)
Supplement: Supplementary file 1 — Appendix S1: Supporting information [file MPR-29-e1834-s001.pdf]

SUPPORTING INFORMATION

Robust Data-Driven Identification of Risk Factors and Their Interactions – A Simulation and a Study of Parental and Demographic Risk Factors for Schizophrenia

Gyllenberg D, McKeague IW, Sourander A, and Brown AS

CONTENTS

LITERATURE SEARCH 1

SUPPLEMENTARY METHODS 2
- **Predictor definitions** 2
- **Detailed rationale for defining the simulation study** 3
- **Simulation study for defining data preprocessing and analyses** 3
- **Simulation study with three active interactions** 3
- **Marginal screening** 4

SUPPLEMENTARY RESULTS 4
- **Simulation study with three active interactions** 4
- **Simulation study using marginal screening** 4
- **Descriptive results of FiPS-S** 4

R-CODE TO REPRODUCE ANALYSES 12
- **Introduction** 12
- **Load functions** 12
- **Simulate data** 12
- **Plot the proportion of predictors** 13
- **Plot the log(OR) between main effect variables** 14
- **Preprocess data** 15
- **Variable selection and assessment of associations** 16
- **Power analyses** 16

RERERENCES 16

LITERATURE SEARCH

Web of Science was searched for prospective studies published between 1998 and 2017 that reported on interactions between parental and demographic risk factors with schizophrenia or related psychoses as an outcome. The following search terms were used:

> *TI=(schizophren* OR psychosis OR psychotic) AND*
> *TS=interaction* AND*
> *TS=(cohort OR prospective OR longitudinal OR follow-up* OR nested OR register OR epidemiol*) AND*
> *TS=(parent* OR famil* OR matern* OR mother OR matern* OR father OR demographic OR urban*) AND*
> *PY=(1998-2017) AND*
> *LANGUAGE:(English) AND*
> *DOCUMENT TYPES:(Article)*

The search resulted in 209 studies. All the titles and abstracts were screened for relevance and full texts were acquired for 64 studies. Among these, 22 studies were prospective, reported on interactions other than gene by environment interactions (reviewed by (Misiak et al., 2018), reported on risk factors available on the individual level and studied diagnosed schizophrenia or non-affective psychoses as outcomes. No restrictions on the methodology to assess interactions were applied. After assessing the full-texts of the 22 studies, two additional studies were identified that fulfilled the objective of the search and that were published between 1998 and 2017 (Harrison et al., 2003; van Os, Hanssen, Bak, Bijl, & Vollebergh, 2003). In total, 24 studies on 35 interactions were summarized in Table 1.

SUPPLEMENTARY METHODS

Predictor definitions

Detailed information about the definition of main effects is shown in Supplemental Table 1.

Supplemental Table 1. Definition of the main effects in the Finnish Prenatal Study of Schizophrenia.

Label	Coding
Sex	0 = female, 1 = male
Born btwn Dec and Mar	0 = birth month between April and November,<br>1 = birth month between December and March
Urbanicity	0 = rural or sub-urban,<br>1 = urban
Parental low education	0 = either parent has post-secondary school education,<br>1 = both parents no post-secondary school education
Parental psychosis	0 = no,<br>1 = yes (ICD-10 codes F20–25, F28–29; ICD-9 codes 295, 297, 298.9X, 301.2C; and ICD-8 codes 295, 297, 298.20, 298.30, 298.99, 299)
Mother 19 or younger	0 = maternal age 20 or older at time of birth of study subject,<br>1 = maternal age 19 or younger at time of birth of study subject
Mother 35 or older	0 = maternal age 34 or younger at time of birth of study subject,<br>1 = maternal age 35 or older at time of birth of study subject
Father 19 or younger	0 = paternal age 20 or older at time of birth of study subject,<br>1 = paternal age 19 or younger at time of birth of study subject
Father 35 or older	0 = paternal age 34 or younger at time of birth of study subject,<br>1 = paternal age 35 or older at time of birth of study subject

Abbreviations: btwn, between; Dec, December; Mar, March; ICD, International Classification of Diseases

Detailed rationale for defining the simulation study

When conducting the simulation study, we aimed at resemblance of epidemiologic data by including groups of variables that were correlated, variables with different prevalence, and both active main and interaction effects. Further, a general assumption is that machine learning techniques can capture complex relationships between variables–such as interactions–with a minimum of a priori restrictions. Therefore, we included an active interaction effect constituted from variables that did not include active main effects, i.e., we applied no hierarchy restriction stipulating that an interaction effect of two variables can only be included in a model if the main effects of the variables are important. Finally, we aimed to optimize the interpretability of the simulation study by setting the active effects from variables with the same prevalence (e.g. all active main effects had the same prevalence) and by not setting multiple active interaction effects taken from the same variables (e.g. if A2 $\times$ B2 is set active, B2 $\times$ C2 should not be set active, because the variable B2 is already included in the other active interaction).

Simulation study for defining data preprocessing and analyses

We performed a simulation study as described in 'Analytic pipeline' in the manuscript, but with varying criteria that defined the maximum absolute log(odds ratio[OR]) between main effects for an interaction to be included in the analyses. Based on 10 000 simulations for each criterion, we calculated the empirical true positive (TP) and false positive (FP) rate for identifying main and interaction effects when using criterion of log(OR) at 0.1, 0.3 and 0.5 (30 000 simulations in total). As shown in Supplemental Table 2, the FP rate remained <5% regardless of the criterion of log(OR). However, the power, i.e. the TP rate, to detect interaction effects was 37.2% when using a log(OR)-criterion of 0.1 compared to 46.7% for a criterion of log(OR) at 0.3 and 45.7% for a criterion of log(OR) at 0.5. Using a strict approach, we opted to use a criterion of log(OR) at 0.3 for the analyses reported in the main text.

As further shown in Supplemental Table 2, we performed a simulation study with varying tuning parameter alpha in the elastic net analyses while keeping the criterion of log(OR) constant at 0.3. This analysis was also based on 10 000 simulations for each value of alpha (0.5, 0.75 and 1). Both the FP and TP rates were similar regardless of which value for the tuning parameter alpha was used. We opted to use an alpha value at 0.75, because the simulations with an alpha value at 0.75 showed the highest TP for detecting interaction effects. A further reason to use an alpha value of 0.75 instead of 1 (i.e. LASSO regression) was based on theory and the literature showing that elastic net analyses with alpha values < 1 perform better in correlated data (Zou & Hastie, 2005).

Simulation study with three active interactions

While the rationale of the primary simulation study reported in the manuscript included several aspects (see 'Detailed rationale for defining the simulation study' above) that were not compatible with setting multiple active interactions of same prevalence in datasets of nine variables, we also conducted an additional simulation study with multiple active interactions but with altered definitions for the simulated datasets. To optimise interpretability and to not set interaction effects taken from variables that were active in other main or interaction effects, we defined simulated datasets as follows: among 4500 subjects, we set the prevalence at 15% for all nine variables (A1, A2 … C3); we set a within-group correlation of 0.3 in the three groups of variables (A, B, C); we set no active main effects; and we set three active interaction effects with an exchangeable OR at 1.5, 2.0, 2.5, 3.0, 3.5, 4.0, 4.5 and 5.0 (A1 $\times$ B1; A2 $\times$ C2; B3 $\times$ C3); these three active interactions were set

to have the same OR in each simulation and were taken between variables in uncorrelated groups having prevalence of 15%, so the interactions had a prevalence of 2.3%.

Marginal screening

We carried out additional simulations to test whether variable selection using elastic net followed by multivariate logistic regression was superior to marginal screening (without prior variable selection). We performed preprocessing of the data as described in 'Analytic pipeline' in the manuscript, but we tested each predictor in a separate model with case-control status as the outcome; models of main effects consisted of only the main effect variable as dependent variable (e.g. formula 'y ~ x1' for testing main effect 'x1') while models of interaction effects consisted the interaction and the two main effect variables included in the interaction (e.g. formula 'y ~ x1 + x2 + x1*x2' for testing interaction 'x1*x2'). We report both results that were and were not Bonferroni-corrected.

SUPPLEMENTARY RESULTS

Simulation study with three active interactions

The FP rates were <5% (Supplemental Table 3) as in the simulation reported in the manuscript. However, the power to detect interactions in these simulated datasets without active main effects were higher than in the simulation in the manuscript which did include three active main effects. For example, to detect interactions with 2.3% prevalence and with >80% power, the OR of the active interaction had to be 5.0 in the simulation reported in the manuscript (Table 2 in the manuscript), while at least one, two or all three active interactions with the same prevalence could be detected at OR=2.0, OR=2.5 and OR=3.5, respectively, in the datasets with no main effects and three active interactions (Supplemental Table 3).

Simulation study using marginal screening

When we carried out marginal screening without Bonferroni-correction, the range of the FP rates were between 47.1% and 91.1% (Supplemental Table 4). When we applied Bonferroni-correction, the TP and FP rates for identifying interactions using marginal screening (Supplemental Table 5) was similar to the corresponding rates using elastic net analyses followed by multivariate logistic regression (Table 2 in the manuscript). However, the FP rate of identifying main effects ranged between 3.6% and 71.1% for all definitions of simulated datasets (Supplemental Table 5).

Descriptive results of FiPS-S

The frequencies and expected counts of all predictors are shown in Supplemental Table 6.

To examine whether the interactions that have been reported in previous studies were roughly consistent with our data, we made additional exploratory analyses of the interactions between parental psychosis and urbanicity, parental psychosis and father 35 or older, and born in winter months and urbanicity. These analyses were performed with marginal screening without Bonferroni-correction and are reported in Supplemental Table 7.

Supplemental Table 2. The true positive (TP) and false positive (FP) rates of identifying main and interactions effects in 10 000 simulated datasets for different data preprocessing criteria and elastic net tuning parameters when using elastic net variable selection and Bonferroni-corrected multivariate logistic regression.

	Criteria in data preprocessing and tuning parameters in analyses		Identification of main effects		Identification of interaction effects	
	log(OR) criterion in data preprocessing	Tuning parameter alpha in elastic net analysis	TP[a] (%)	FP[b] (%)	TP[c] (%)	FP[d] (%)
Primary definition of simulated datasets	0.3	0.75	57.3	1.3	46.6	4.5
Varying log(OR) criterion in data preprocessing						
	0.1	0.75	63.6	2.5	37.2	4.0
	0.5	0.75	56.8	1.1	45.7	4.3
Varying tuning parameter in data preprocessing						
	0.3	0.50	56.4	1.1	45.7	3.6
	0.3	1.00	59.2	1.3	46.2	4.2

Abbreviations: TP, true positive; FP, false positive.
Note: The simulation datasets were in all analyses defined with 4500 subjects, 2.3% prevalence of the active interaction, OR=3 of the active interaction and a 0.3 within-group correlation.
[a] The TP rate of identifying main effects was defined as the proportion of simulations in which at least one of the three active main effects were correctly identified.
[b] The FP rate of identifying main effects was defined as the proportion of simulations in which at least one of the non-active main effects were incorrectly identified.
[c] The TP rate of identifying interaction effects was defined as the proportion of simulations in which the one active interaction effect was correctly identified.
[d] The FP rate of identifying interaction effects was defined as the proportion of simulations in which at least one of the non-active interactions effects were incorrectly identified.

Supplemental Table 3. The true positive (TP) and false positive (FP) rates of identifying interaction effects in 10 000 simulated datasets using elastic net variable selection and Bonferroni-corrected multivariate logistic regression. The simulated datasets were set to have 4500 subjects, a prevalence of 15% among all variables, no active main effects, a within-group correlation of 0.3 and an exchangeable OR of the three active interactions that were taken between variables in uncorrelated groups. All three active interactions had the same OR in each simulation.

	Identification of main effects	Identification of interaction effects			
OR of active interactions	FP[a] (%)	FP[b] (%)	TP for at least 1 interaction[c] (%)	TP for at least 2 interactions[d] (%)	TP for all 3 interactions[e] (%)
1.5	2.4	4.4	22.9	2.1	0.1
2.0	1.9	3.5	**82.8**	43.3	10.2
2.5	1.8	2.7	**98.5**	**87.0**	48.0
3.0	1.5	2.1	**99.8**	**97.1**	74.9
3.5	1.8	2.1	**100.0**	**99.2**	**86.1**
4.0	1.7	1.7	**100.0**	**99.6**	**90.1**
4.5	1.4	1.6	**100.0**	**99.6**	**92.5**
5.0	1.5	1.7	**100.0**	**99.7**	**93.4**

Abbreviations: TP, true positive; FP, false positive.

Note: Simulated datasets with the TP rate ≥ 80 % are shown in bold.

[a] The proportion of simulations in which at least one (non-active) main effect were incorrectly identified.

[b] The proportion of simulations in which at least one of the non-active interactions effects were incorrectly identified.

[c] The proportion of simulations in which at least one of the three active interaction effects were correctly identified.

[d] The proportion of simulations in which at least two of the three active interaction effects were correctly identified.

[e] The proportion of simulations in which all three active interaction effects were correctly identified.

Supplemental Table 4. The true positive (TP) and false positive (FP) rates of identifying main and interactions effects in 10 000 simulated datasets using marginal screening without Bonferroni-correction.

	Definition of simulated datasets				Identification of main effects		Identification of interaction effects	
	OR of active interaction	No. of subjects	Prevalence of active interaction (%)	Within-group correlation	TP[a] (%)	FP[b] (%)	TP[c] (%)	FP[d] (%)
Primary definition of simulated datasets	3.0	4500	2.3	0.3	99.8	71.5	91.1	66.2
Varying OR of active interaction								
	1.5	4500	2.3	0.3	99.7	47.3	26.8	64.5
	2.0	4500	2.3	0.3	99.8	58.4	60.3	64.8
	2.5	4500	2.3	0.3	99.8	66.3	82.4	65.4
	3.5	4500	2.3	0.3	99.8	76.2	95.8	66.3
	4.0	4500	2.3	0.3	99.8	78.4	97.6	66.1
	4.5	4500	2.3	0.3	99.8	81.2	98.4	65.8
	5.0	4500	2.3	0.3	99.8	82.3	99.0	66.4
Varying number of subjects								
	3.0	9000	2.3	0.3	100.0	92.9	99.8	72.3
	3.0	15000	2.3	0.3	100.0	99.1	100.0	77.5
Varying prevalence of active interaction								
	3.0	4500	2.9	0.3	99.8	80.8	96.1	67.4
	3.0	4500	4.0	0.3	99.8	91.0	99.0	67.8
Varying within-group correlation								
	3.0	4500	2.3	0.1	99.7	58.9	92.2	70.6
	3.0	4500	2.3	0.5	99.9	85.3	89.8	67.8

Abbreviations: TP, true positive; FP, false positive. Note: Simulated datasets with both the TP rate ≥ 80 % and the FP rate < 5 % are shown in bold. [a] The TP rate of identifying main effects was defined as the proportion of simulations in which at least one of the three active main effects were correctly identified; [b] The FP rate of identifying main effects was defined as the proportion of simulations in which at least one of the non-active main effects were incorrectly identified; [c] The TP rate of identifying interaction effects was defined as the proportion of simulations in which the one active interaction effect was correctly identified; [d] The FP rate of identifying interaction effects was defined as the proportion of simulations in which at least one of the non-active interactions effects were incorrectly identified.

Supplemental Table 5. The true positive (TP) and false positive (FP) rates of identifying main and interactions effects in 10 000 simulated datasets using Bonferroni-corrected marginal screening.

	Definition of simulated datasets				Identification of main effects		Identification of interaction effects	
	OR of active interaction	No. of subjects	Prevalence of active interaction (%)	Within-group correlation	TP[a] (%)	FP[b] (%)	TP[c] (%)	FP[d] (%)
Primary definition of simulated datasets	3.0	4500	2.3	0.3	83.8	12.7	43.2	2.5
Varying OR of active interaction								
	1.5	4500	2.3	0.3	**82.8**	**3.6**	1.9	2.7
	2.0	4500	2.3	0.3	83.8	6.7	10.6	2.4
	2.5	4500	2.3	0.3	83.8	9.3	27.3	2.4
	3.5	4500	2.3	0.3	84.0	15.2	56.3	2.1
	4.0	4500	2.3	0.3	83.8	18.4	66.3	2.7
	4.5	4500	2.3	0.3	83.9	19.3	74.2	2.6
	5.0	4500	2.3	0.3	84.7	21.9	**80.8**	**2.5**
Varying number of subjects								
	3.0	9000	2.3	0.3	99.9	37.3	**92.7**	**3.0**
	3.0	15000	2.3	0.3	100.0	71.1	**99.9**	**3.9**
Varying prevalence of active interaction								
	3.0	4500	2.9	0.3	83.9	20.0	61.8	2.4
	3.0	4500	4.0	0.3	84.9	34.2	**82.6**	**2.6**
Varying within-group correlation								
	3.0	4500	2.3	0.1	80.1	6.8	44.5	2.4
	3.0	4500	2.3	0.5	87.2	22.3	37.8	2.9

Abbreviations: TP, true positive; FP, false positive. Note: Simulated datasets with both the TP rate ≥ 80 % and the FP rate < 5 % are shown in bold. [a] The TP rate of identifying main effects was defined as the proportion of simulations in which at least one of the three active main effects were correctly identified; [b] The FP rate of identifying main effects was defined as the proportion of simulations in which at least one of the non-active main effects were incorrectly identified; [c] The TP rate of identifying interaction effects was defined as the proportion of simulations in which the one active interaction effect was correctly identified; [d] The FP rate of identifying interaction effects was defined as the proportion of simulations in which at least one of the non-active interactions effects were incorrectly identified.

Supplemental Table 6. Frequencies and expected counts of predictors by case-control status.

Predictor	Controls (n=2975) No. (%)	Controls (n=2975) Expected count	Cases (n=1469) No. (%)	Cases (n=1469) Expected count
Predictors with expected counts ≥ 5 in both cases and controls				
Male sex	1712 (57.5)	1707.7	839 (57.1)	843.3
Born btwn Dec and Mar	994 (33.4)	994.8	492 (33.5)	491.2
Urbanicity	1649 (55.4)	1719.1	919 (62.6)	848.9
Parental low education	305 (10.3)	332.0	191 (13.0)	164.0
Parental psychosis	91 (3.1)	212.9	227 (15.5)	105.1
Mother 19 or younger	98 (3.3)	103.1	56 (3.8)	50.9
Mother 35 or older	369 (12.4)	391.6	216 (14.7)	193.4
Father 19 or younger	28 (0.9)	28.1	14 (1.0)	13.9
Father 35 or older	665 (22.4)	705.6	389 (26.5)	348.4
Male sex x Born btwn Dec and Mar	601 (20.2)	602.5	299 (20.4)	297.5
Male sex x Urbanicity	938 (31.5)	983.4	531 (36.1)	485.6
Male sex x Parental low education	171 (5.7)	180.7	99 (6.7)	89.3
Male sex x Parental psychosis	60 (2.0)	119.2	118 (8.0)	58.8
Male sex x Mother 19 or younger	58 (1.9)	58.2	29 (2.0)	28.8
Male sex x Mother 35 or older	213 (7.2)	219.6	115 (7.8)	108.4
Male sex x Father 19 or younger	14 (0.5)	14.7	8 (0.5)	7.3
Male sex x Father 35 or older	387 (13.0)	401.0	212 (14.4)	198.0
Born btwn Dec and Mar x Urbanicity	551 (18.5)	586.4	325 (22.1)	289.6
Born btwn Dec and Mar x Parental low education	103 (3.5)	112.5	65 (4.4)	55.5
Born btwn Dec and Mar x Parental psychosis	33 (1.1)	75.0	79 (5.4)	37.0
Born btwn Dec and Mar x Mother 19 or younger	31 (1.0)	33.5	19 (1.3)	16.5
Born btwn Dec and Mar x Mother 35 or older	138 (4.6)	139.2	70 (4.8)	68.8
Born btwn Dec and Mar x Father 35 or older	236 (7.9)	243.7	128 (8.7)	120.3

Supplemental Table 6. Continued.

Predictor	Controls (n=2975)		Cases (n=1469)	
	No. (%)	Expected count	No. (%)	Expected count
Urbanicity x Parental low education	176 (5.9)	195.5	116 (7.9)	96.5
Urbanicity x Parental psychosis	46 (1.5)	125.9	142 (9.7)	62.1
Urbanicity x Mother 19 or younger	55 (1.8)	62.3	38 (2.6)	30.7
Urbanicity x Mother 35 or older	192 (6.5)	214.9	129 (8.8)	106.1
Urbanicity x Father 19 or younger	17 (0.6)	16.1	7 (0.5)	7.9
Urbanicity x Father 35 or older	349 (11.7)	380.9	220 (15.0)	188.1
Parental low education x Parental psychosis	13 (0.4)	33.5	37 (2.5)	16.5
Parental low education x Mother 19 or younger	16 (0.5)	26.1	23 (1.6)	12.9
Parental low education x Mother 35 or older	61 (2.1)	58.9	27 (1.8)	29.1
Parental low education x Father 35 or older	115 (3.9)	117.8	61 (4.2)	58.2
Parental psychosis x Mother 35 or older	13 (0.4)	31.5	34 (2.3)	15.5
Parental psychosis x Father 35 or older	18 (0.6)	52.9	61 (4.2)	26.1
Mother 19 or younger x Father 19 or younger	17 (0.6)	16.7	8 (0.5)	8.3
Mother 35 or older x Father 35 or older	300 (10.1)	306.6	158 (10.8)	151.4
Predictors with expected counts < 5 in either cases or controls				
Born btwn Dec and Mar x Father 19 or younger	10 (0.3)	10.0	5 (0.3)	5.0[a]
Parental low education x Father 19 or younger	4 (0.1)	7.4	7 (0.5)	3.6
Parental psychosis x Mother 19 or younger	3 (0.1)	10.0	12 (0.8)	5.0[a]
Parental psychosis x Father 19 or younger	2 (0.1)	4.0	4 (0.3)	2.0
Mother 19 or younger x Father 35 or older	2 (0.1)	2.0	1 (0.1)	1.0
Mother 35 or older x Father 19 or younger	0 (0)	0.0	0 (0)	0.0

[a] The expected count is < 5 but rounded to 5.0.

Supplemental Table 7. Marginal screening of interaction effects reported in previous studies and the corresponding descriptive results of analyses including a mutually exclusive four-class variable.

	Controls (n=2975)	Cases (n=1469)		
	No. (%)	No. (%)	OR	Non-corrected p-value of interaction term
Model including main effects and interaction term				
Parental psychosis	91 (3.1)	227 (15.5)	5.2	
Urbanicity	1649 (55.4)	919 (62.6)	1.3	
Parental psychosis x Urbanicity	46 (1.5)	142 (9.7)	1.2	0.44
Model including one four-class variable				
No parental psychosis and Rural	1281 (43.1)	465 (31.7)	1	
No parental psychosis and Urban	1603 (53.9)	777 (52.9)	1.3	
Parental psychosis and Rural	45 (1.5)	85 (5.8)	5.2	
Parental psychosis and Urban	46 (1.5)	142 (9.7)	8.5	
Model including main effects and interaction term				
Parental psychosis	91 (3.1)	227 (15.5)	5.6	
Father 35 or older	665 (22.4)	389 (26.5)	1.2	
Parental psychosis x Father 35 or older	18 (0.6)	61 (4.2)	1.2	0.56
Model including one four-class variable				
No parental psychosis and Father 34 or younger	2237 (75.2)	914 (62.2)	1	
No parental psychosis and Father 35 or older	647 (21.7)	328 (22.3)	1.2	
Parental psychosis and Father 34 or younger	73 (2.5)	166 (11.3)	5.6	
Parental psychosis and Father 35 or older	18 (0.6)	61 (4.2)	8.3	
Model including main effects and interaction term				
Born btwn Dec and Mar	994 (33.4)	492 (33.5)	0.9	
Urbanicity	1649 (55.4)	919 (62.6)	1.2	
Born btwn Dec and Mar x Urbanicity	551 (18.5)	325 (22.1)	1.3	0.10
Model including one four-class variable				
Born btwn Apr and Nov and Rural	883 (29.7)	383 (26.1)	1	
Born btwn Apr and Nov and Urban	1098 (36.9)	594 (40.4)	1.2	
Born btwn Dec and Mar and Rural	443 (14.9)	167 (11.4)	0.9	
Born btwn Dec and Mar and Urban	551 (18.5)	325 (22.1)	1.4	
Model restricted to subjects born btwn Apr and Nov				
Rural	883 (44.6)	383 (39.2)	1	
Urban	1098 (55.4)	594 (60.8)	1.3	
Model restricted to subjects born btwn Dec and Mar				
Rural	443 (44.6)	167 (33.9)	1	
Urban	551 (55.4)	325 (66.1)	1.6	

Abbreviations: btwn, between; Dec, December; Mar, March; Apr, April; Nov, November; OR, odds ratio

R-CODE TO REPRODUCE ANALYSES

Introduction

The functions and the code described in the repository rely on the following R-packages: tidyverse (Wickham, 2017), simstudy (Goldfeld, 2018), stringr (Wickham, 2018), glmnet (Friedman, Hastie, & Tibshirani, 2010), furrr (Vaughan, 2018), tictoc (Izrailev, 2014), openxlsx (Walker, 2018) and knitr (Xie, 2018).

Load functions

Clone the repository from *https://github.com/davgyl/dd_ident* (DOI: 10.5281/zenodo.3778351) and load the functions from the R-scripts.

```
source("01_load_pkgs.R")
source("02_simdata.R")
source("03_preprocess.R")
source("04_plot_prev.R")
source("05_plot_heatmap.R")
source("06_glmnet.R")
```

Simulate data

Use the `dd_sim`-function to produce simulation data.

```
# Apply function and set seed for reproducible example
data <- dd_sim(seed = 1000)
# Display data
data

## # A tibble: 4,500 x 10
##        y    A1    A2    A3    B1    B2    B3    C1    C2    C3
##    <dbl> <dbl> <dbl> <dbl> <dbl> <dbl> <dbl> <dbl> <dbl> <dbl>
##  1     0     0     0     0     0     0     0     0     0     0
##  2     0     0     0     0     0     0     0     0     0     0
##  3     0     0     0     0     0     0     0     0     0     0
##  4     1     0     0     0     1     0     0     1     1     0
##  5     0     1     1     0     0     0     0     0     0     0
##  6     0     0     0     0     0     0     0     1     0     0
##  7     0     0     0     0     0     0     0     0     0     0
##  8     0     0     0     0     1     1     0     0     0     0
##  9     1     0     0     0     0     0     0     1     0     0
## 10     0     0     0     0     0     1     0     0     0     0
## # ... with 4,490 more rows
```

Plot the proportion of predictors

Use the `dd_plot_prop`-function to the proportion of predictors by case-control status. Note that main effects `A1`, `B1` and `C1` are active at OR=1.3 and interaction `A2 x B2` is active at OR=3.

```
dd_plot_prop(data)
```

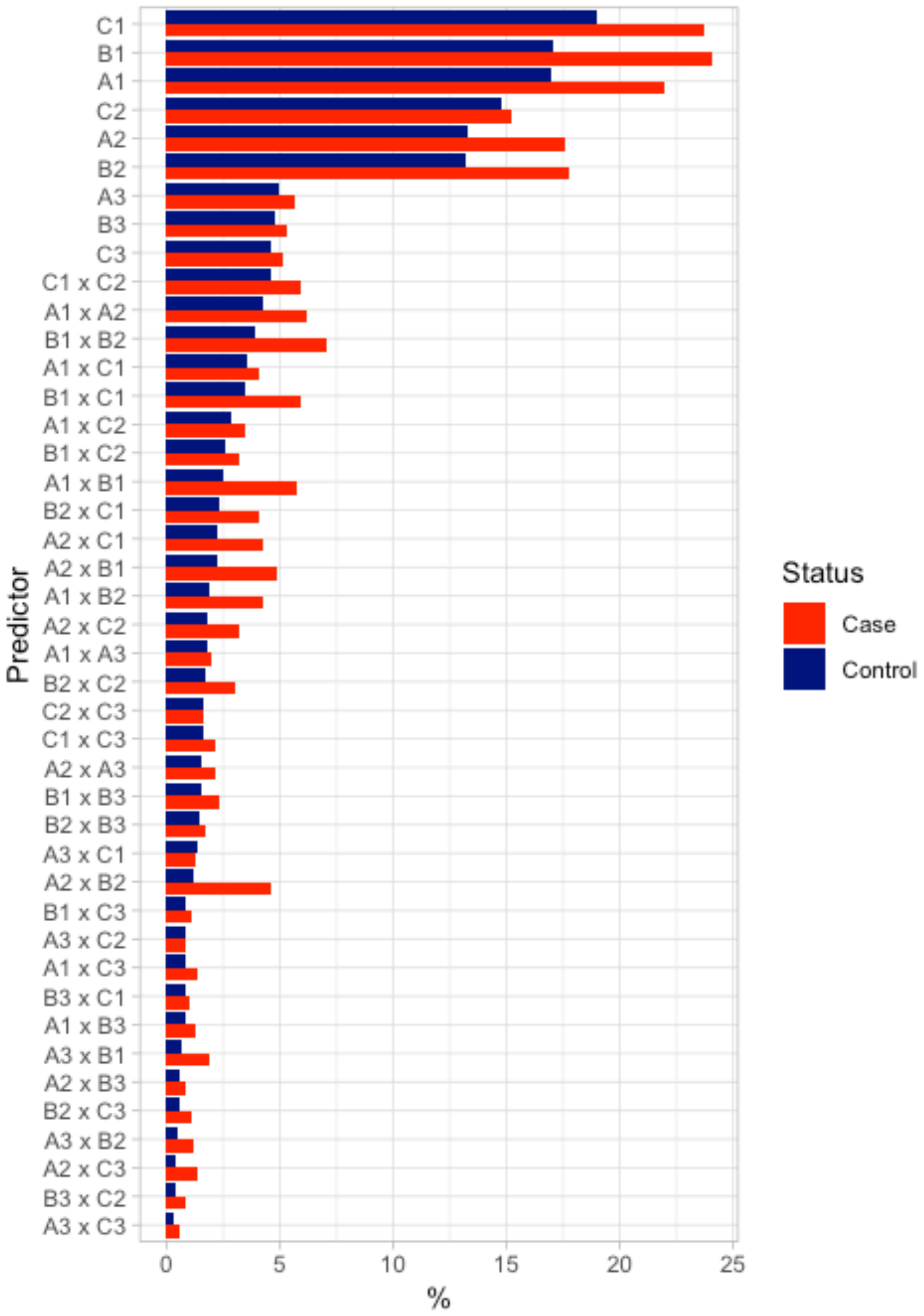

Plot the log(OR) between main effect variables

Note that the three groups of variables (A, B, C) are defined to have a within-group correlation in the simulated data.

```
dd_heatmap(data, limit = c(-4, 4))
```

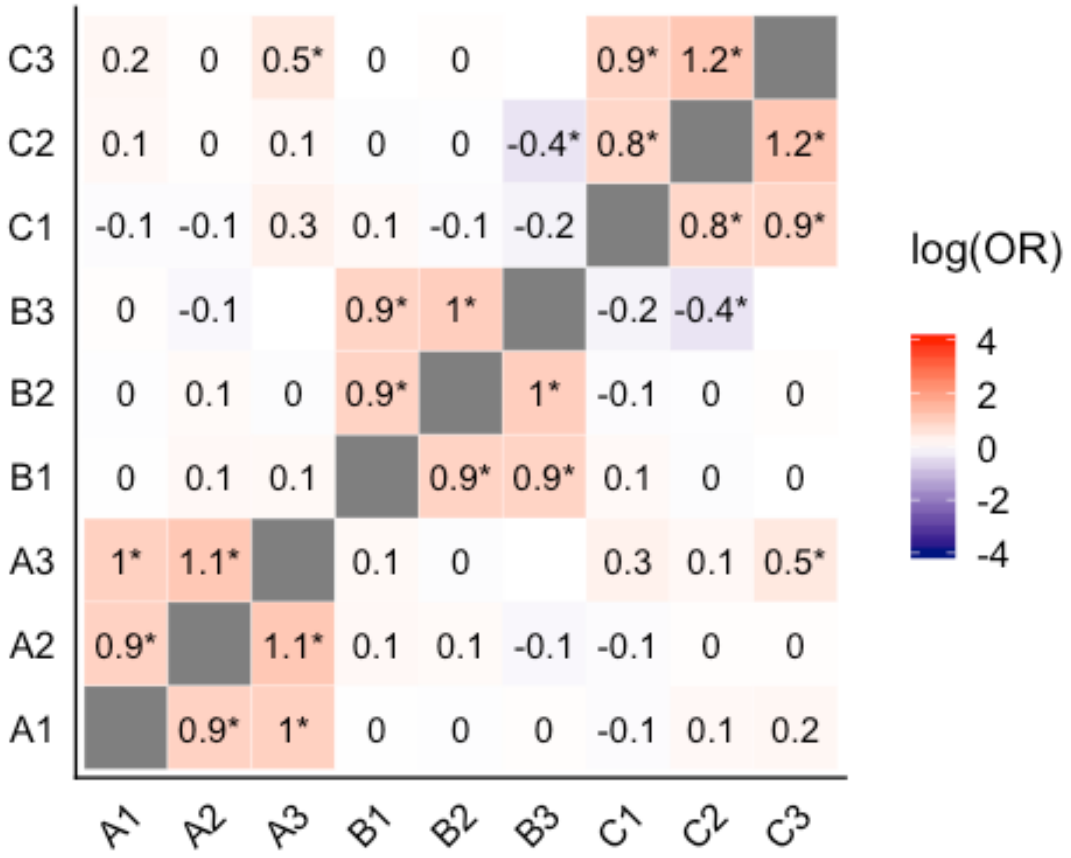

Preprocess data

Use the *dd_preprocess*-function to preprocess data as described in the manuscript. This will produce a list-object containing three dataframes:
- The frequencies, prevalence (%) and expected count of predictors
- The correlational structure of the main effects
- The preprocessed data with included predictors (main and interaction effects)

```
proc_data <-
  data %>%
  dd_preprocess()
```

Display the preprocessed data.

```
proc_data$data

## # A tibble: 4,500 x 34
##        y    A1    A2    A3    B1    B2    B3    C1    C2    C3 A1_x_B1
##    <dbl> <dbl> <dbl> <dbl> <dbl> <dbl> <dbl> <dbl> <dbl> <dbl>   <dbl>
##  1     0     0     0     0     0     0     0     0     0     0       0
##  2     0     0     0     0     0     0     0     0     0     0       0
##  3     0     0     0     0     0     0     0     0     0     0       0
##  4     1     0     0     0     1     0     0     1     1     0       0
##  5     0     1     1     0     0     0     0     0     0     0       0
##  6     0     0     0     0     0     0     0     1     0     0       0
##  7     0     0     0     0     0     0     0     0     0     0       0
##  8     0     0     0     0     1     1     0     0     0     0       0
##  9     1     0     0     0     0     0     0     1     0     0       0
## 10     0     0     0     0     0     1     0     0     0     0       0
## # ... with 4,490 more rows, and 23 more variables: A1_x_B2 <dbl>,
## #   A1_x_B3 <dbl>, A1_x_C1 <dbl>, A1_x_C2 <dbl>, A1_x_C3 <dbl>,
## #   A2_x_B1 <dbl>, A2_x_B2 <dbl>, A2_x_B3 <dbl>, A2_x_C1 <dbl>,
## #   A2_x_C2 <dbl>, A2_x_C3 <dbl>, A3_x_B1 <dbl>, A3_x_B2 <dbl>,
## #   A3_x_C1 <dbl>, A3_x_C2 <dbl>, B1_x_C1 <dbl>, B1_x_C2 <dbl>,
## #   B1_x_C3 <dbl>, B2_x_C1 <dbl>, B2_x_C2 <dbl>, B2_x_C3 <dbl>,
## #   B3_x_C1 <dbl>, B3_x_C2 <dbl>
```

Variable selection and assessment of associations

Use the *dd_select*-function to perform variable selection with the elastic net and assess the Bonferroni-corrected p-values and confidence intervals (CI) of the sparse model as described in the manuscript.

In the simulation data, the main effects *A1*, *B1* and *C1* are defined as active at OR=1.3 and the interaction *A2 x B2* is defined active at OR=3.

```
set.seed(1000)
dd_select(proc_data$data)$bonf %>%
  kable
```

Predictor	OR	low_ci	high_ci	p
A1	1.27	0.95	1.68	0.291
B1	1.39	1.05	1.83	0.006
C1	1.32	1.04	1.69	0.009
A1_x_B1	1.42	0.76	2.68	1.000
A2_x_B2	3.58	1.87	7.16	0.000

For a more exploratory approach, the significance level of the confidence intervals can be increased.

```
set.seed(1000)
dd_select(proc_data$data, sign_level = 0.1)$bonf %>%
  kable
```

Predictor	OR	low_ci	high_ci	p
A1	1.27	0.97	1.65	0.291
B1	1.39	1.07	1.80	0.006
C1	1.32	1.05	1.66	0.009
A1_x_B1	1.42	0.79	2.57	1.000
A2_x_B2	3.58	1.95	6.83	0.000

Power analyses

To conduct power analyses, modify and run the *08_power_repeat_sim.R*-script and summarize the results as described in the *09_summarize_sim_res.R*-script.

RERERENCES

Friedman, J., Hastie, T., & Tibshirani, R. (2010). Regularization Paths for Generalized Linear Models via Coordinate Descent. *Journal of Statistical Software, 33*(1), 1-22.

Goldfeld, K. (2018). simstudy: Simulation of Study Data. R package version 0.1.10. Retrieved from *https://CRAN.R-project.org/package=simstudy*

Harrison, G., Fouskakis, D., Rasmussen, F., Tynelius, P., Sipos, A., & Gunnell, D. (2003). Association between psychotic disorder and urban place of birth is not mediated by obstetric complications or childhood socio-economic position: a cohort study. *Psychological Medicine, 33*(4), 723-731.

Izrailev, S. (2014). tictoc: Functions for timing R scripts, as well as implementations of Stack and List structures. R package version 1.0. Retrieved from *https://CRAN.R-project.org/package=tictoc*

Misiak, B., Stramecki, F., Gaweda, L., Prochwicz, K., Sasiadek, M. M., Moustafa, A. A., & Frydecka, D. (2018). Interactions Between Variation in Candidate Genes and Environmental Factors in the Etiology of Schizophrenia and Bipolar Disorder: a Systematic Review. *Mol Neurobiol, 55*(6), 5075-5100. doi:10.1007/s12035-017-0708-y

van Os, J., Hanssen, M., Bak, M., Bijl, R. V., & Vollebergh, W. (2003). Do urbanicity and familial liability coparticipate in causing psychosis? *The American Journal of Psychiatry, 160*(3), 477-482. doi:10.1176/appi.ajp.160.3.477

Vaughan, D. D., M. (2018). furrr: Apply Mapping Functions in Parallel using Futures. R package version 0.1.0. Retrieved from *https://CRAN.R-project.org/package=furrr*

Walker, A. (2018). openxlsx: Read, Write and Edit XLSX Files. R package version 4.1.0. Retrieved from *https://CRAN.R-project.org/package=openxlsx*

Wickham, H. (2017). tidyverse: Easily Install and Load the 'Tidyverse'. R package version 1.2.1. Retrieved from *https://CRAN.R-project.org/package=tidyverse*

Wickham, H. (2018). stringr: Simple, Consistent Wrappers for Common String Operations. R package version 1.3.1. Retrieved from *https://CRAN.R-project.org/package=stringr*

Xie, Y. (2018). knitr: A General-Purpose Package for Dynamic Report Generation in R. R package version 1.20. Retrieved from *https://cran.r-project.org/web/packages/knitr/index.html*

Zou, H., & Hastie, T. (2005). Regularization and variable selection via the elastic net. *Journal of the Royal Statistical Society Series B-Statistical Methodology, 67*, 301-320. doi:DOI 10.1111/j.1467-9868.2005.00503.x
